# Effects of different enantiomers of amlodipine on lipid profiles and vasomotor factors in atherosclerotic rabbits

**DOI:** 10.1515/biol-2021-0077

**Published:** 2021-09-06

**Authors:** Jing Zhang, Ming-yan Yao, Guo-rui Zhang, Xian-ru Chen, Qi Liu, Yifang Guo, Xin-wei Jia

**Affiliations:** Department of Cardiology, Affiliated Hospital of Hebei University, Baoding, Hebei, 071000, China; Department of Endocrinology, Baoding No. 1 Central Hospital, Baoding, Hebei, 071000, China; Department of Cardiology, The Third Hospital of Shijiazhuang, Shijiazhuang, Hebei, 050000, China; Department of Cardiology, Affiliated Hospital of Hebei Engineering University, Handan, Hebei, 056000, China; Department of Cardiology, Shijiazhuang Traditional Chinese Medicine Hospital, Shijiazhuang, Hebei, 050000, China; Cardiology Division in Geriatric Institute, Hebei Provincial People’s Hospital, Shijiazhuang, Hebei, 050000, China

**Keywords:** atherosclerosis, R-amlodipine, S-amlodipine, VECs

## Abstract

This research aimed to describe the functions of vascular endothelial cells (VECs) in protecting target organs and the anti-atherosclerotic effects of different enantiomers of amlodipine on a rabbit model of atherosclerosis. Thirty male New Zealand white rabbits were randomly allocated to four groups (nA = 9, nB = 7, nC = 7, and nD = 7 rabbits): rabbits in group-A (control group) were fed a high-fat diet, group-B rabbits were fed a high-fat diet plus 2.5 mg/kg/day S-amlodipine, group-C rabbits were fed a high-fat diet plus 2.5 mg/kg/day R-amlodipine, and group-D rabbits were fed a high-fat diet plus 5 mg/kg/day racemic amlodipine. Different enantiomers of amlodipine did not influence lipid profiles and serum level of eNOS in the rabbit atherosclerosis model but decreased ET-1 expression to some extent. The serum NO and iNOS levels in the drug intervention groups were significantly reduced. No significant differences in the rabbits’ body weights were observed. At the 4th and 8th weeks, the serum lipid profiles significantly increased in high cholesterol diet groups. The serum ET-1 level was significantly increased in each group of rabbits at the 8th week. Both S-amlodipine and R-amlodipine may protect the endothelium by reducing the serum ET-1 level, downregulating iNOS expression.

## 1 Introduction

Calcium channel blockers (CCBs), also known as calcium antagonists (CA), constitute a category of hypotensive drugs widely applied in clinics for many years. CCBs are widely used to treat hypertension, chronic coronary ischemia, and/or supraventricular arrhythmias. Previous studies alluding to the increased cardiovascular risk associated with CCBs use have been silenced by an array of outcomes trials that show these drugs to be both safe and effective in reducing hard cardiovascular endpoints [1]. Furthermore, according to the 2007 European Society of Hypertension (ESH)/European Society of Cardiology (ESC) guidelines for the treatment of hypertension, CCBs have added beneficial anti-atherosclerotic effects on coronary and carotid vessels, independent of their abilities to reduce blood pressure [2].

Amlodipine, a long-acting dihydropyridine-type CCB, is a chiral compound composed of an S-enantiomer of amlodipine (S-amlodipine) and an R-enantiomer of amlodipine (R-amlodipine) in the same ratio. Amlodipine and l-amlodipine are common hypotensive drugs used in clinics in China. The activity of the S-enantiomer of amlodipine, which accounts for the primary cardiovascular activity, is 1,000-fold greater than the activity of R-amlodipine in reducing blood pressure [3]. In addition, according to basic research, amlodipine exerts a beneficial effect on atherosclerosis in animals. Clinical research studies, such as the classical PREVENT and CAMELOT studies, have shown that CCBs have an anti-atherosclerosis effect. Long-term treatment with CCBs may decrease the carotid intima-media thickness (IMT) in hypertensive patients and attenuate the progression of atherosclerosis [4,5]. Combination therapies of amlodipine with other agents causing blockade of the renin–angiotensin–aldosterone pathway (angiotensin II receptor blockers or renin inhibitors) have shown successful blood pressure reduction approaches to decrease the risk of CV and progression of renal disease. The new form of CCBs has been established that have additional properties of blocking calcium channel subtypes T and N and exert particular action on heart rate and renin aldosterone system apart from their class effects. Because of these additional features, they are considered to be more renoprotective [6].

In recent years, researchers have proposed that R-amlodipine may have the same anti-atherosclerosis effects as S-amlodipine, but only one *in vitro* study revealed that R-amlodipine induces the release of nitric oxide (NO) from the coronary artery in the canine species. Therefore, we speculate that R-amlodipine may function to retard the pathogenesis of atherosclerosis and protect target organs by regulating vascular endothelial function [7]. However, currently, there is a lack of studies that prove that R-amlodipine may function to retard the pathogenesis of atherosclerosis. Therefore, studies determining whether an essential difference exists between amlodipine and l-amlodipine in the protection of target organs will be very crucial. This study aims to describe the functions of vascular endothelial cells (VECs) in protecting target organs and the anti-atherosclerotic effects of different enantiomers of amlodipine on a rabbit model of atherosclerosis.

## 2 Materials and methods

### 2.1 Animals

Thirty male New Zealand white rabbits (parent generation, F_0_) (2.0 ± 0.3 kg) were obtained from the Laboratory Animal Center of Hebei Medical University. Rabbits were housed in an animal room in Hebei Provincial People’s Hospital and provided free access to rabbit chow and tap water.

**Ethical approval:** The research related to animal use has been complied with all the relevant national regulations and institutional policies for the care and use of animals. The research conforms with the Guide for the Care and Use of Laboratory Animals published by the US National Institutes of Health (NIH Publication No. 85-23, revised 1996). The study protocol was approved by the Animal Ethics Committee of Hebei Medical University (K2015-019-1).

### 2.2 Treatments

**Figure 1:**
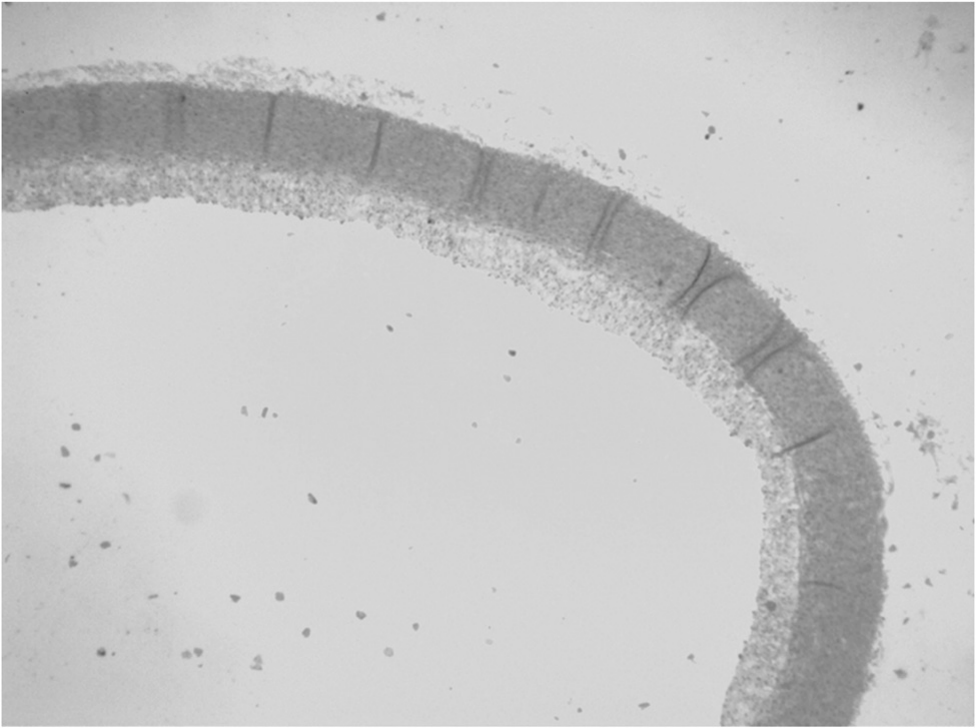
Plaques from the control group were observed under a light microscope at the 4th week. The models were successfully established.

**Figure 2:**
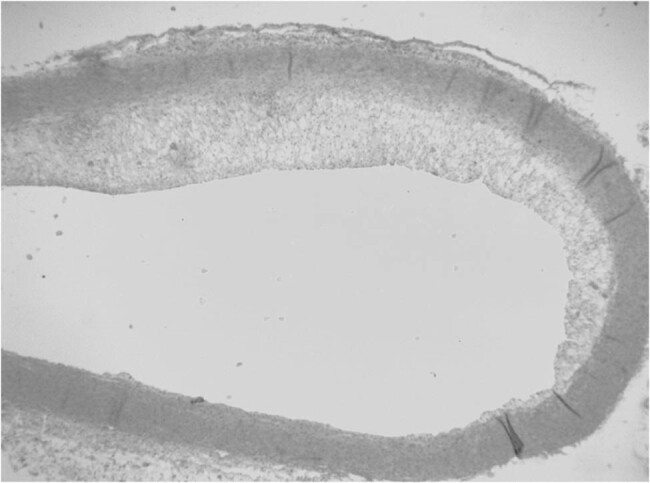
Plaques from the control group were observed under a light microscope at the 8th week. The models were successfully established.

The rabbits were randomly allocated to four groups (nA = 9, nB = 7, nC = 7, and nD = 7 rabbits): rabbits in group A (control group) were fed a high-fat diet (containing 1% pure cholesterol and 1% edible lard), rabbits in group B were fed a high-fat diet plus 2.5 mg/kg/day S-amlodipine, rabbits in group C were fed a high-fat diet plus 2.5 mg/kg/day R-amlodipine, and rabbits in group D were fed a high-fat diet plus 5 mg/kg/day racemic amlodipine. Drugs were dissolved in distilled water and administered via a special gavage tube at 08:00 every morning. All rabbits were allowed a 1-week adaptation period before the study and were then fed the respective diet protocols for 8 weeks. One rabbit from group A was euthanized at each of the 4th and 8th week, and a 5 mm length of the distal aortic arch was removed. Aortas were fixed with formaldehyde for 24 h, embedded in paraffin, sliced into pathological sections, and stained with standard hematoxylin–eosin dyes (H&E). The pathological changes in the HE-stained specimens were observed under a light microscope. Models were confirmed to be successfully established by forming an atherosclerotic plaque (Figures 1 and 2).

### 2.3 Drugs

Amlodipine and S-amlodipine powders were provided by CSPC Ouyi Pharmaceutical Co., Ltd. (Shijiazhuang, Hebei, China). The R-amlodipine powder was provided by Shihuida Pharma Group (Jilin, China). The cholesterol powder was produced by Qitian Chemical Industry Co., Ltd. (Nanjing, Jiangsu, China). Edible lard was available in the market.

### 2.4 Measurement of lipid profiles and vasomotor factors

Total cholesterol (TC), low-density lipoprotein cholesterol (LDL-C), high-density lipoprotein cholesterol (HDL-C), and triglycerides (TG) were included in the lipid profiles in this study. Vasomotor factors, including NO, nitric oxide synthase (NOS), and endothelin-1 (ET-1), were also evaluated in this study. At weeks 0, 4, and 8, all animals were fasted for 12–14 h and blood was drawn from the ear vein to measure the lipid profiles and the vasomotor factors. Blood was allowed to stand for 1 hour before centrifugation at 3,000 rpm for 15 min, and the separated serum samples were then stored at −70°C until assay in batches.

### 2.5 Plasma lipoproteins and biochemical determinations

Blood was drawn in the nonfasting state with a 20-gauge Teflon catheter introduced into the central artery of the ear (Vasofix, B. Braun) into tubes containing disodium EDTA to a final concentration. TC, LDL-C, HDL-C, and TG levels were measured with an automatic biochemistry analyzer (HORIBA, pentra C400).

Plasma NO concentrations were measured using a Nitric Oxide Assay Kit (Nanjing Jiancheng Bioengineering Institute), as described in the study by Deng et al. [8]. Plasma NOS concentrations were measured using a Nitric Oxide Synthase Typed Assay Kit (Nanjing Jiancheng Bioengineering Institute), as described in the study by Deng et al. [8].

### 2.6 Plasma ET-1 levels

Plasma ET-1 levels were measured with a Rabbit Endothelin-1 ELISA Kit (Shanghai Mingjing Biology). Briefly, all reagents and samples were prepared. Then, 100 µL of a standard or a sample were added to each well, followed by a 2 h incubation at 37°C. The samples were aspirated, and 100 µL of the prepared Detection Reagent A was added and incubated for 1 h at 37°C. The samples were then aspirated, washed 3 times, and 100 µL of the prepared Detection Reagent B was added and incubated for 1 h at 37°C. The samples were again aspirated and washed 5 times. Then, 90 µL of the Substrate Solution was added and incubated for 15–25 min at 37°C. Finally, 50 µL of the Stop Solution was added, and the absorbance of the samples was immediately measured at 450 nm.

### 2.7 Statistical analysis

SPSS22.0 software for Windows was used for the statistical analysis. Data are presented as the mean ± standard deviation (SD) and were analyzed using one-way ANOVA when a normal distribution and the homogeneity of variance were satisfied. When the data were not normally distributed, the nonparametric Kruskal–Wallis test was used. Inter-group differences were assessed using the Student–Newman–Keuls (SNK) q test for comparisons between two groups. *p*-values <0.05 were considered statistically significant.

## 3 Results

### 3.1 Animals

Thirty male New Zealand white rabbits were adaptively fed for 1 week and all proceeded to the drug intervention stage. With the exception of the two rabbits from the control group that was euthanized at the 4th and 8th weeks, the remaining rabbits survived to the end of this experiment. No unexpected deaths occurred during the process of animal feeding.

### 3.2 Bodyweight

After 8 weeks of feeding on a high cholesterol diet, the body weights of each group of rabbits increased significantly. No significant differences in the rabbits’ body weights were observed among the 4 groups at baseline (*p* = 0.22), the 4th week (*p* = 0.31), or the 8th week (*p* = 0.34) (Table 1).

**Table 1:** Average body weight of rabbits at baseline, the 4th week, and the 8th week

Group	*n*	Baseline (kg)	4th week (kg)	8th week (kg)
A	9/8/7	2.03 ± 0.17	3.12 ± 0.21	3.55 ± 0.30
B	7	2.08 ± 0.20	3.09 ± 0.27	3.62 ± 0.34
C	7	2.12 ± 0.21	3.13 ± 0.30	3.58 ± 0.28
D	7	2.06 ± 0.26	3.07 ± 0.31	3.51 ± 0.29

Note: Data are presented as mean ± SD. *p* < 0.05 was considered a significant difference. Group A = high-fat diet alone (control group), group B = high-fat diet plus 2.5 mg/kg/day S-amlodipine, group C = high-fat diet plus 2.5 mg/kg/day R-amlodipine, and group D = high-fat diet plus 5 mg/kg/day racemic amlodipine. *p* > 0.05 when the drug groups (groups B, C, and D) were compared with group A.

### 3.3 Serum lipid profiles

At the 4th and 8th weeks, the serum lipid profiles (TC, LDL-C, HDL-C, and TG) were significantly increased in the groups of rabbits that were fed the high cholesterol diets compared with the baseline values. A high cholesterol diet induced hypercholesterolemia in our rabbit model. However, statistically, significant differences were not observed between the groups at each feeding stage (Tables 2–4), indicating that the different enantiomers of amlodipine did not influence the lipid profiles (TC, LDL-C, HDL-C, and TG).

**Table 2:** Effects of different enantiomers of amlodipine on serum TC, LDL-C, HDL-C, and TG levels in rabbits at baseline

Group	*n*	TC (mmol/L)	LDL-C (mmol/L)	HDL-C (mmol/L)	TG (mmol/L)
A	9	3.97 ± 0.45	1.03 ± 0.19	1.41 ± 0.41	0.77 ± 0.31
B	7	3.84 ± 0.61	1.02 ± 0.22	1.18 ± 0.24	0.68 ± 0.23
C	7	4.20 ± 0.99	1.11 ± 0.27	1.30 ± 0.39	0.74 ± 0.37
D	7	3.85 ± 0.75	1.08 ± 0.26	1.46 ± 0.61	0.84 ± 0.21

Data are presented as mean ± SD. *p* < 0.05 was considered a significant difference. Group A = high-fat diet alone (control group), group B = high-fat diet plus 2.5 mg/kg/day S-amlodipine, group C = high-fat diet plus 2.5 mg/kg/day R-amlodipine, and group D = high-fat diet plus 5 mg/kg/day racemic amlodipine. TC = total cholesterol, LDL-C = low-density lipoprotein cholesterol, HDL-C = high-density lipoprotein cholesterol, TG = triglyceride. *p* > 0.05 when the drug groups (group B, C, and D) were compared with group A.

**Table 3:** Effects of different enantiomers of amlodipine on serum TC, LDL-C, HDL-C, and TG levels in rabbits at the 4th week

Group	*n*	TC (mmol/L)	LDL-C (mmol/L)	HDL-C (mmol/L)	TG (mmol/L)
A	8	11.97 ± 2.28	6.41 ± 0.93	3.66 ± 0.62	1.91 ± 0.34
B	7	11.14 ± 1.46	5.85 ± 1.39	2.64 ± 0.81	1.85 ± 0.43
C	7	10.71 ± 2.45	6.09 ± 1.58	3.70 ± 1.32	1.83 ± 0.22
D	7	11.64 ± 2.86	5.27 ± 0.79	2.91 ± 0.67	1.77 ± 0.47

Data are presented as mean ± SD. *p* < 0.05 was considered a significant difference. Group A = high-fat diet alone (control group), group B = high-fat diet plus 2.5 mg/kg/day S-amlodipine, group C = high-fat diet plus 2.5 mg/kg/day of R-amlodipine, and group D = high-fat diet plus 5 mg/kg/day racemic amlodipine. TC = total cholesterol, LDL-C = low-density lipoprotein cholesterol, HDL-C = high-density lipoprotein cholesterol, TG = triglyceride. *p* > 0.05 when the drug groups (group B, C, and D) were compared with group A.

**Table 4:** Effects of different enantiomers of amlodipine on serum TC, LDL-C, HDL-C, and TG levels in rabbits at the 8th week

Group	*n*	TC (mmol/L)	LDL-C (mmol/L)	HDL-C (mmol/L)	TG (mmol/L)
A	7	15.23 ± 2.68	8.77 ± 1.86	4.91 ± 0.70	3.05 ± 0.28
B	7	13.66 ± 2.88	7.81 ± 1.69	3.82 ± 0.96	3.19 ± 0.60
C	7	14.04 ± 3.45	8.12 ± 1.27	4.12 ± 1.25	2.95 ± 0.61
D	7	14.75 ± 1.82	7.24 ± 1.57	4.54 ± 0.82	3.21 ± 0.56

Data are presented as mean ± SD. Differences of *p* < 0.05 were considered significant. Group A = high-fat diet alone (control group), group B = high-fat diet plus 2.5 mg/kg/day S-amlodipine, group C = high-fat diet plus 2.5 mg/kg/day R-amlodipine, and group D = high-fat diet plus 5 mg/kg/day racemic amlodipine. TC = total cholesterol, LDL-C = low-density lipoprotein cholesterol, HDL-C = high-density lipoprotein cholesterol, TG = triglyceride. *p* > 0.05 when the drug groups (groups B, C, and D) were compared with group A.

### 3.4 Serum vasomotor factors

#### 3.4.1 Baseline levels of vasomotor factors

**Figure 3:**
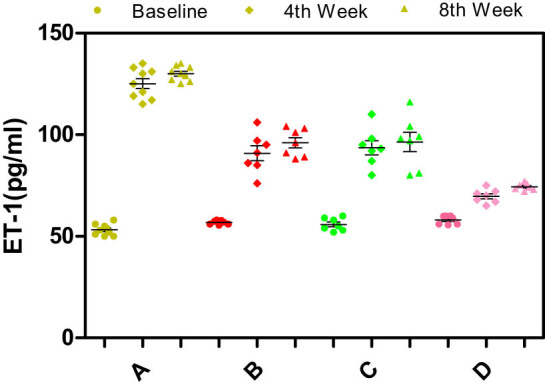
Effects of different enantiomers of amlodipine on the serum ET-1 levels in rabbits at baseline, the 4th week, and the 8th week. Data are presented as mean ± SD. *p* < 0.05 was considered a significant difference. Group A = high-fat diet alone (control group), group B = high-fat diet plus 2.5 mg/kg/day S-amlodipine, group C = high-fat diet plus 2.5 mg/kg/day R-amlodipine, and group D = high-fat diet plus 5 mg/kg/day racemic amlodipine. ET-1 = endothelin-1. ^*^ *p* > 0.05 compared with group A, ^#^ *p* < 0.01 compared with group A, and ^$^ *p* < 0.01 compared with group B or C.

**Figure 4:**
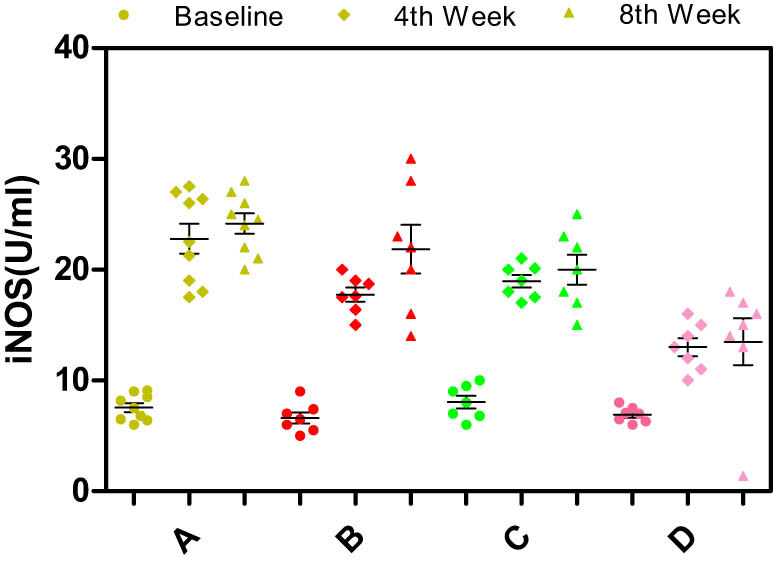
Effects of different enantiomers of amlodipine on serum iNOS levels in rabbits at baseline, the 4th week, and the 8th week. Data are presented as mean ± SD. *p* < 0.05 was considered a significant difference. Group A = high-fat diet alone (control group), group B = high-fat diet plus 2.5 mg/kg/day S-amlodipine, group C = high-fat diet plus 2.5 mg/kg/day R-amlodipine, and group D = high-fat diet plus 5 mg/kg/day racemic amlodipine. NO = nitric oxide. ^*^ *p* < 0.01, ^#^ *p* < 0.01, and ^$^ *p* < 0.01 compared with group A.

**Figure 5:**
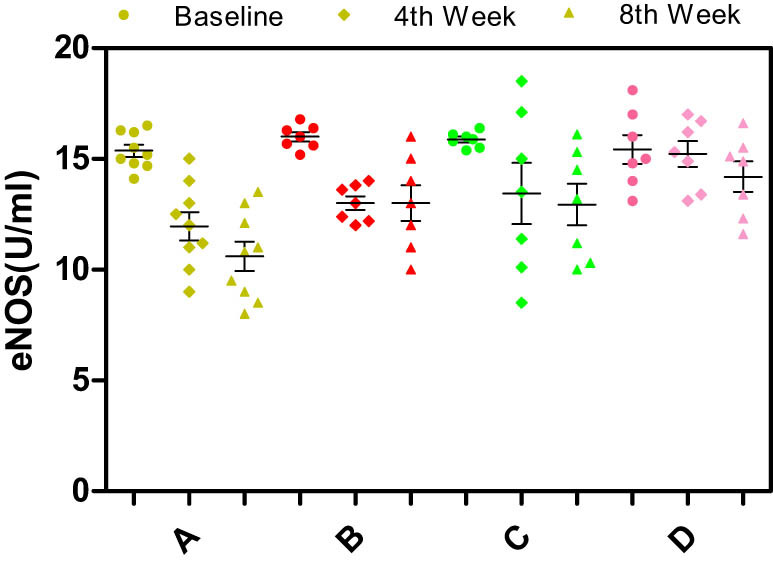
Effects of different enantiomers of amlodipine on serum eNOS levels in rabbits at baseline, the 4th week, and the 8th week. Data are presented as mean ± SD. *p* < 0.05 was considered a significant difference. Group A = high-fat diet alone (control group), group B = high-fat diet plus 2.5 mg/kg/day S-amlodipine, group C = high-fat diet plus 2.5 mg/kg/day R-amlodipine, and group D = high-fat diet plus 5 mg/kg/day racemic amlodipine. iNOS = inducible nitric oxide synthase. ^*^ *p* < 0.01, ^#^ *p* < 0.01, and ^$^ *p* < 0.01 compared with group A.

**Figure 6:**
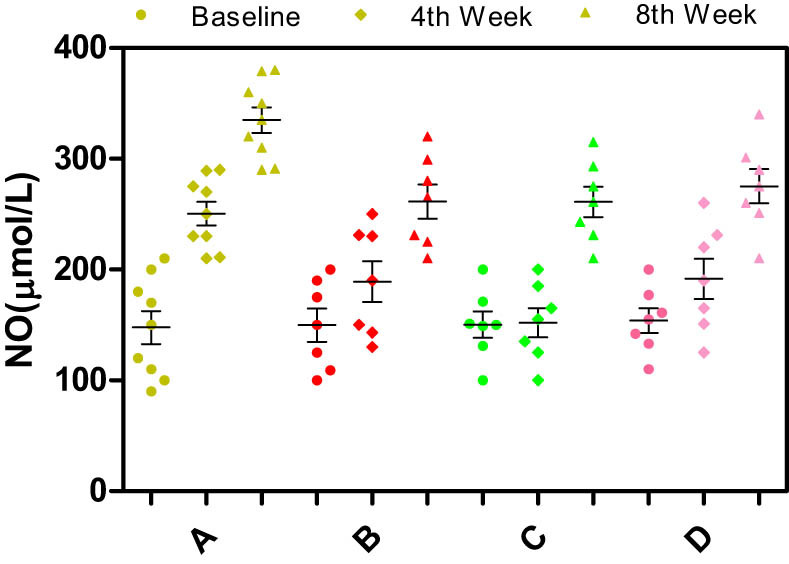
Effects of different enantiomers of amlodipine on serum NO levels in rabbits at baseline, the 4th week, and the 8th week. Data are presented as mean ± SD. *p* < 0.05 was considered a significant difference. Group A = high-fat diet alone (control group), group B = high-fat diet plus 2.5 mg/kg/day S-amlodipine, group C = high-fat diet plus 2.5 mg/kg/day R-amlodipine, and group D = high-fat diet plus 5 mg/kg/day racemic amlodipine. eNOS = endothelial nitric oxide synthase. ^*^ *p* > 0.05, ^#^ *p* > 0.05, and ^$^ *p* > 0.05 compared with group A.

Significant differences in the serum levels of NO, ET-1, endothelial nitric oxide synthase (eNOS), or inducible nitric oxide synthase (iNOS) were not observed among the different groups of rabbits at baseline (*p* > 0.05) (Figures 3–6).

#### 3.4.2 Levels of vasomotor factors at the 4th and 8th weeks

The serum ET-1 level was significantly increased in each group of rabbits at the 8th week. Serum ET-1 levels were lower in the drug intervention groups than in the control group at the 4th and 8th weeks. The serum ET-1 level was much lower in group D (5 mg/kg/day racemic amlodipine) than in group B (2.5 mg/kg/day S-amlodipine), or group C (2.5 mg/kg/day R-amlodipine) (*p* < 0.01). No significant differences were observed between groups B and C (*p* > 0.05) (Figure 3), indicating that different enantiomers of amlodipine downregulate ET-1 expression to some extent.

Serum levels of NO and iNOS: As time progressed, the serum levels of NO and iNOS obviously increased. Compared with the control group, NO levels in the drug intervention groups decreased significantly at the 4th and 8th weeks (*p* < 0.01). No significant differences in the serum levels of NO were observed among groups B, C, and D (*p* > 0.05) (Figure 4). The serum iNOS levels were significantly decreased in the drug intervention groups at the 4th and 8th weeks compared with those in the control group (*p* < 0.01). Moreover, the serum iNOS level in group D showed a decreasing trend compared with those in groups B and C. No significant differences in iNOS levels were observed among groups B, C, and D (*p* > 0.05) (Figure 5). Based on these results, S- and R-amlodipine may downregulate the expression of NO and iNOS.

Serum level of eNOS: The serum eNOS levels showed an increasing trend as the feeding time increased. However, statistically significant differences were not observed between the control group and the drug intervention groups at the 4th and 8th weeks (*p* > 0.05) (Figure 6), suggesting that the different enantiomers of amlodipine did not affect the serum eNOS levels in the rabbit atherosclerosis model.

## 4 Discussion

Due to their important roles in maintaining the physiological function of the human circulatory system, VECs are present throughout human tissues and are located in the epithelial lining of capacitance vessels, resistance vessels, and capillaries. In addition to serving as a mechanical barrier, VECs also represent an important paracrine organ. Endothelium vasoactive substances (VECs) secrete many vasoactive substances, such as NO, ET-1, thromboxane A-2 (TXA-2), angiotensin II (Ang II), and prostacyclin (PGI-2) [9,10,11]. The maintenance of vasomotor function, the regulation of angiogenesis and cell proliferation, inflammatory immune responses, alterations in vascular permeability, the balance between coagulation and fibrinolysis, and the suppression of the accumulation and adhesion of inflammatory cells all depend on neurohumoral regulation by VECs. NO and ET-1 are important vasoactive substances. The dynamic balance between these substances has an important role in maintaining the good physiological function of blood vessels. However, the imbalance may lead to vasomotor dysfunction, which is also closely related to hypertension [12,13]. Angiotensin-converting enzyme (ACE) expression ultimately leads to the constriction of vascular smooth muscle cells (VSMCs) by converting Ang I to Ang II, inducing the tyrosine phosphorylation of eNOS, reducing NO synthesis, and promoting ET-1 production [14]. ET-1 is similar to growth factors in some respects and promotes the formation of early atherosclerotic lesions by inducing the proliferation of arterial smooth muscle cells [15]. ET-1 levels contribute to the hypertensive atherosclerotic process and dramatically influence the risks of cardiovascular and cerebrovascular diseases [16,17]. The combination of antihypertensive therapy with the drugs amlodipine and lisinopril is highly effective in correcting endothelial dysfunction by increasing the NO level and decreasing the ET-1 level in hypertensive patients with type 2 diabetes mellitus (T2DM) [18]. In addition, the ET-1 level is a valuable independent predictor of coronary artery calcification (CAC), which is considered one of the important predictors of atherosclerosis [19]. Conversely, NO exerts anti-atherosclerosis effects through various mechanisms, such as inhibiting adhesion between endothelial cells and monocytes [20]. Amlodipine treatments may ameliorate endothelial dysfunction through their antioxidant activity and improve NO availability in spontaneously hypertensive rats [21]. VECs constitute the target organ of cardiovascular diseases and risk factors. Vascular endothelial dysfunction is closely related to the development of diseases and unhealthy lifestyles. Based on the current basic and clinical research, vascular endothelial dysfunction may be one of the most important initial steps in atherosclerosis [22].

The formation of atherosclerotic plaques is very complicated. Many factors, such as blood vessel cells, the extracellular matrix, blood components, hemodynamics, the *in vivo* environment, and hereditary factors, are related to atherosclerosis. In addition, large arteries, including the coronary artery, the cerebral artery, the renal artery, and the lower extremity artery, are often involved in atherosclerosis, but arterioles are rarely eroded. Decreased levels of NO and eNOS are often observed in patients with hypertension, indicating that endothelial dysfunction (ED) and hypertension often co-exist [23]. CCBs have been shown to inhibit the formation of atherosclerotic plaques independent of their hypotensive effect. According to the results of the INSIGHT study, nifedipine not only delays the progression of CAC but also can reverse the thickening of the carotid artery intima-media [24]. Lacidipine exerted better effects than atenolol on attenuating carotid artery intima-media thickness in the ELSA study [25]. Nifedipine was recently shown to prevent endothelial cell senescence in an eNOS-dependent manner, potentially representing a novel mechanism by which this drug protects against atherosclerosis [26]. Amlodipine is a long-acting dihydropyridine-type CCB and is a racemic mixture of S-amlodipine and R-amlodipine. Amlodipine inhibits Ang II-induced aortic aneurysms and atherosclerosis in hypercholesterolemic mice [27]. Amlodipine increases NO release by modulating the eNOS/Caveolin-1 interaction [28]. Combined treatment with angiotensin receptor blockers (ARBs) and amlodipine markedly prevent atherosclerotic lesion development and progression in a model of atherosclerosis [29,30]. In addition to its primary effect on blood pressure, amlodipine delays the progression of atherosclerosis, but the mechanism underlying this additional effect remains unclear. According to the results of an *in vitro* study of canine species, R-amlodipine increases NO release [31]. The anti-atherosclerotic effect of amlodipine is presumed to benefit from the R-enantiomer by stimulating NO generation.

Dyslipidemia is generally increased LDL-C or TG levels or decreased HDL-C levels in serum. Because it injures endothelial cells, dyslipidemia is an important risk factor for atherosclerosis. Serum lipids are components of atherosclerosis because they deposit on vessel walls through several pathways. The accumulation of excess levels of these lipids will lead to lipid plaque formation, which is called atherosclerosis. Low-density lipoprotein (LDL) is a carrier that delivers cholesterol from the liver to all body tissues. As shown in the study by Ogasswara et al., serum LDL levels are associated with atherosclerosis and coronary artery disease (CAD) [32]. LDL increases platelet activity, promoting the progression of atherosclerosis [33]. High-density lipoprotein (HDL) has a role in reverse cholesterol transport, carrying cholesterol from peripheral tissues back to the liver via the scavenger receptor β-1. Accordingly, this transport may protect the cardiovascular system [34]. HDL elicits a broad spectrum of biological responses, such as improving endothelial function, increasing NO and prostacyclin release, and inhibiting platelet aggregation and apoptosis [35]. Moreover, HDL/LDL has an advantage over LDL alone [36,37]. Besides, according to the results of the PROCAM study, hypertriglyceridemia is an independent risk factor for CAD [38]. Increased TG levels and decreased HDL-C levels reflect the primary dyslipidemia phenotype, which is an element in the residual risk of vascular events in patients with cardiovascular disease [39].

Different enantiomers of amlodipine had no effect on lipid profiles (TC, LDL-C, HDL-C, and TG) in the experimental groups compared with those in the control group at weeks 0, 4, and 8. In theory, amlodipine is a hypotensive drug but not a lipid-lowering agent. However, the short feeding time and very high serum lipid levels may have distorted the test outcomes and even led to false-negative results. Nevertheless, in this study, the serum lipid levels of each group were at an equivalent level.

VECs play an important role in human physiological functions, and normal vascular endothelial function is vitally important in preventing cardiovascular disease. ED is one of the key steps in the formation of early atherosclerotic lesions. VECs secrete vasoactive factors that regulate vasomotor function at the physiological level and conditions that upset this balance may cause vascular ED. NO is produced from l-arginine, which is catalyzed to l-citrulline by eNOS [40]. Vasodilation dysfunction is a primary cause of ED and is induced by the decreased synthesis and release of NO [41]. The pro-inflammatory state generated by dyslipidemia, which decreases NOS and NO production, produces ED [42]. ET-1 is a powerful endothelium-derived contracting factor (EDCF) secreted by VECs that induces VSMC contraction by stimulating the calcium channel and accelerating Ca^2+^ exchange. Conditions that interrupt the dynamic equilibrium between ET-1 and NO in serum may also cause ED. A decrease in NO bioactivity may cause a relative increase in ET-1 levels, leading to vascular remodeling, vasoconstriction, and dysfunction [43]. Furthermore, an ET-1 receptor antagonist was shown to improve the vascular endothelial function of a patient with coronary heart disease (CHD), confirming that ET-1 plays an important role in ED and the development of atherosclerosis in the early stage to some extent [44]. Three different NOS isoforms have been identified in mammals, including eNOS, neuronal nitric oxide synthase (nNOS), and iNOS. Both eNOS and nNOS are constitutive nitric oxide synthases (cNOS). As nNOS is primarily expressed in the nervous system, our research does not cover this isoform. eNOS is often synthesized by the vascular endothelium and exerts important anti-atherosclerosis effects through multiple pathways by promoting the catalytic synthesis of NO. Serum eNOS contributes to the regulation of blood pressure and nitrite homeostasis [45]. iNOS is mainly expressed in macrophages, mastocytes, and neutrophils. It is not expressed in resting cells but is more frequently expressed in cells stimulated with endotoxins, neurokines, and immunity-inducing substances. This reaction causes a sequence of pathological effects by producing large amounts of NO. Upregulation of iNOS produces additional NO and promotes nitrosative stress and ED. The abnormal iNOS activity increases arginase activity and competes with eNOS for l-arginine, thus resulting in the reduced bioavailability of physiological NO [46]. A selective iNOS inhibitor has been shown to delay the progression of atherosclerosis by reducing the production of pathological NO [47].

As feeding time increased, the serum ET-1 level increased significantly. This phenomenon may be related to severe atherosclerosis and endothelial dysfunction in rabbits fed with a high-fat diet. The serum ET-1 level was lower in the experimental groups than in the control group at the 4th and 8th weeks, and the serum level in group D was much lower than the levels observed in groups B and C. The antihypertensive effect of amlodipine mainly results from its S-enantiomer. Groups B and D received the same dose of S-amlodipine in the daily feed, which may have theoretically caused a similar decrease in blood pressure in these groups. Based on the results of our study, different enantiomers of amlodipine decrease ET-1 expression to some extent, but the results from groups B and D may be affected by changes in blood pressure, suggesting that the hypotensive effect of S-amlodipine may lead to positive results. Group C also displayed positive results, and group D exhibited a larger decrease in ET-1 levels than group B. In some sense, R-amlodipine indeed decreased ET-1 expression when it did not cause changes in blood pressure usually.

As time progressed, the serum NO and iNOS levels increased substantially. The same reason for the increase of ET-1 level, the pathological process may lead to iNOS activation and a large number of pathological NO release. Compared with the control group, the levels of NO in the drug intervention groups were significantly decreased at the 4th and 8th weeks. However, significant differences were not observed among groups B, C, and D. The serum iNOS levels in the drug intervention groups were significantly reduced. Moreover, group D showed a continuous decreasing trend compared with groups B and C. However, significant differences were not observed among groups B, C, and D. This may be due to the small sample size of the study. If the sample size increases, it may show a statistical difference. Thus, S- and R-amlodipine may decrease NO and iNOS expression. The serum eNOS levels exhibited a gradually increasing tendency as the feeding time increased, but statistically, differences were not observed between the control group and the drug intervention groups. The serum level of eNOS in the drug intervention groups showed an increasing trend compared with the control group. It may be due to that a decrease of serum ET-1 level in drug intervention groups may promote eNOS expression. Different enantiomers of amlodipine had no effect on the level of eNOS in the rabbit atherosclerosis model in this study but may have reduced ET-1 and iNOS expression and inhibited NO release.

In conclusion, different enantiomers of amlodipine protect the endothelium by reducing the serum ET-1 level, down-regulating iNOS expression, and inhibiting the release of pathological NO.

## 5 Conclusions

(1) Different enantiomers of amlodipine had no effect on lipid profiles (TC, LDL-C, HDL-C, and TG) in the rabbit atherosclerosis model.
(2) S-amlodipine and R-amlodipine may protect the endothelium by reducing the serum ET-1 level, downregulating iNOS expression, and inhibiting the release of pathological NO. The enantiomers may exert an anti-atherosclerosis effect. Our study also found that enantiomers of amlodipine had no effect on eNOS expression in the rabbit atherosclerosis model.

There is some limitation to this study. Severe atherosclerosis may lead to the activation of iNOS and the release of excess pathological NO, which exerts the complete opposite biological functions to physiological NO [38]. The atherosclerotic lesion is very serious in our model; therefore, the effects of the excess pathological NO may be far greater than the effects of physiological NO. We did not distinguish these two kinds of NO, and thus the amount of pathological NO may have concealed the variation in pathological NO in this research. Further studies are required to study this effect in a larger animal model. Theoretically, amlodipine is a hypotensive drug, but not a lipid-lowering agent. However, the short feeding time and very high serum lipid levels may have distorted the test outcomes and even led to false-negative results. However, in this study, the serum lipid levels of each group were at an equivalent level. This effect needs to be elaborated further *in vitro* and *in vivo*.
